# Imaging of built-in electric field at a *p-n* junction by scanning transmission electron microscopy

**DOI:** 10.1038/srep10040

**Published:** 2015-06-12

**Authors:** Naoya Shibata, Scott D. Findlay, Hirokazu Sasaki, Takao Matsumoto, Hidetaka Sawada, Yuji Kohno, Shinya Otomo, Ryuichiro Minato, Yuichi Ikuhara

**Affiliations:** 1 Institute of Engineering Innovation, School of Engineering, The University of Tokyo, Yayoi 2-11-16, Bunkyo-ku, Tokyo 113-8656, Japan; 2 Japan Science and Technology Agency, PRESTO, 4-1-8 Honcho Kawaguchi, Saitama 332-0012, Japan; 3 School of Physics and Astronomy, Monash University, Victoria 3800, Australia; 4 Furukawa Electric Ltd., 2-4-3 Okano, Nishi-ku, Yokohama, 220-0073, Japan; 5 JEOL Ltd., 1-2-3 Musashino, Akishima, Tokyo 196-8558, Japan; 6 Nanostructures Research Laboratory, Japan Fine Ceramic Center, 2-4-1 Mutsuno, Atsuta-ku, Nagoya 456-8587, Japan

## Abstract

Precise measurement and characterization of electrostatic potential structures and the concomitant electric fields at nanodimensions are essential to understand and control the properties of modern materials and devices. However, directly observing and measuring such local electric field information is still a major challenge in microscopy. Here, differential phase contrast imaging in scanning transmission electron microscopy with segmented type detector is used to image a *p-n* junction in a GaAs compound semiconductor. Differential phase contrast imaging is able to both clearly visualize and quantify the projected, built-in electric field in the *p-n* junction. The technique is further shown capable of sensitively detecting the electric field variations due to dopant concentration steps within both *p*-type and *n*-type regions. Through live differential phase contrast imaging, this technique can potentially be used to image the electromagnetic field structure of new materials and devices even under working conditions.

Differential phase contrast (DPC) imaging has been used in many microscopy techniques such as optical, X-ray and electron microscopy^1^,^2^,^3^,^4^,^5^. DPC images are formed by taking the difference between the signals recorded on detector segments diametrically opposed about the optical axis, and thereby map variations in beam deflection and/or asymmetric intensity redistribution in the diffraction plane as the probe is scanned across the specimen. In scanning transmission electron microscopy (STEM), DPC has been used to image local magnetic structures in magnetic materials at low-to-medium resolution^6^,^7^,^8^,^9^. In recent years, DPC STEM imaging has been extended to the characterization of local electric field distributions in polar and ferroelectric materials by detecting the Coulomb deflection of the incident electron beam^10^,^11^,^12^. The development of a segmented-type annular detector suitable for atomic-resolution STEM has further enabled DPC imaging at atomic-resolution^11^. In the present study we apply DPC STEM to visualize the built-in electric field formed at a *p*-*n* junction in a GaAs compound semiconductor, even though the simultaneous bright-field (BF) or annular dark-field (ADF) images do not visualize the *p-n* junction. *p*-*n* junctions are one of the most important electronic interfaces in electronic devices, and the precise characterization of their position and structure is in high demand. While electron holography and Lorentz transmission electron microscopy (TEM) are currently used for such tasks, the capability shown here to characterize the local electrostatic potential structures and associated electric fields will be of benefit to the field of semiconductor device characterization, particularly as it can be carried out simultaneously with other STEM imaging modes with established strengths for structure and composition analysis.

Unlike TEM or Fresnel mode in Lorentz TEM, where imposing large defocus or other lens aberrations is essential to make the phase shifts imparted by the sample visible in the recorded intensity^13^, optimal DPC imaging occurs at the in-focus condition^14^. This makes DPC imaging compatible with standard STEM imaging modes like ADF, which can then be used simultaneously to provide further information for structural characterization. DPC imaging can be easily operated in STEM using suitable segmented-type detectors and is compatible with the wide range of established STEM imaging and analytical capabilities. Moreover, DPC STEM images can be monitored as so-called “live images” — i.e. at the few-second-long refresh rate of the scan during the experiment — without any complicated post-acquisition processing (see supporting movie for the live imaging of the *p-n* junction). DPC STEM can thus be applied to the electromagnetic field structure of new materials and devices not only as a tool for analysis but as a tool for discovery.

## Results

**Figure 1:**
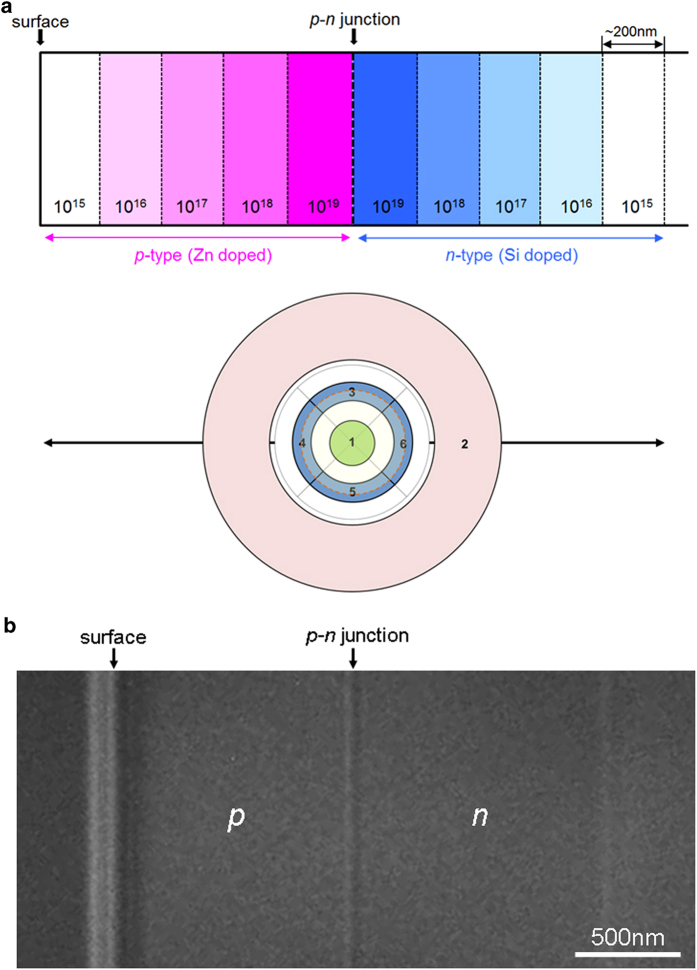
Schematic illustration and Lorentz TEM image of the GaAs semiconductor sample observed in this study. (**a**) Schematic illustration of the GaAs semiconductor *p-n* junction sample and its orientation relationship with respect to the detector segments. (**b**) A Lorentz TEM image of the *p-n* junction region imaged at an under-defocus condition of 0.7 mm.

Figure 1a shows the schematic illustration of the GaAs semiconductor sample and its orientation relationship with respect to the detector segments. The alignment of the opposing detector segments 4 and 6 is in the direction perpendicular to the *p*-*n* junction plane. Note that in this sample compositional steps of donors and acceptors are present within the *n*-type and *p*-type regions, respectively. Electrostatic potential steps should be formed at these compositional steps, though the corresponding electric fields will be much smaller than the built-in electric field of the *p-n* junction. Observation of these steps is therefore an excellent test of the sensitivity of the current DPC STEM system. By way of reference, we simultaneously observe two STEM images with circular and ring detector geometries. Labeled 1 and 2 in the schematic and with angle ranges of 0–57 μrad and 224–422 μrad, these correspond to BF and low-angle ADF imaging conditions, respectively. Fig. 1b shows a Lorentz TEM image of the GaAs sample in the highly under-focused condition of 0.7 mm. Line contrast is seen in this image at the position of the *p*-*n* junction, confirming its presence in the present sample.

**Figure 2:**
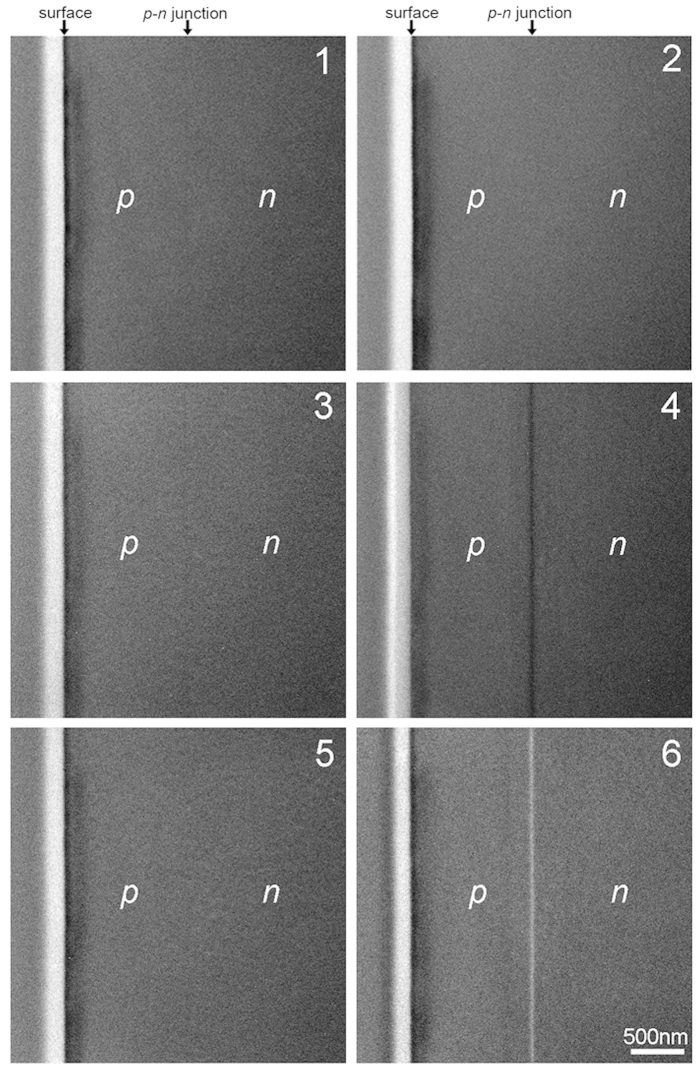
Simultaneous STEM images observed by the segmented detector. Simultaneous STEM images of the *p-n* junction region formed by the respective detector segments (numbered 1 through 6) shown in Fig. 1a.

Figure 2 show the simultaneously obtained segmented detector images from the *p-n* junction region in the GaAs sample. The numbers on each image correspond to the detector segments defined in Fig.1. In the present experiment, we observed the GaAs sample in-focus, near to the [110] orientation. The sample was tilted by a degree or two off-axis to maximize the DPC contrast while minimizing bend contours—another advantage of live imaging. The former constraint reflects the fact that the plane of the *p*-*n* junction and hence its maximum field need not lie exactly in the [110] direction. The latter reflects the need to minimize dynamical diffraction effects which might otherwise lead to a non-uniform bright field intensity distribution purely through elastic scattering processes (see the supplemental material for a detailed discussion on this point). The *p*-type and *n*-type regions are labelled in the figures, and the *p-n* junction should be present between them. However, the images from segments 1 and 2, the more conventional STEM imaging modes, show no significant contrast at the *p-n* junction region: the sample appears to be a uniform single crystal. Thus, it is exceedingly difficult to visualize the *p-n* junction in in-focus STEM images using circular or ring detector geometry. Images 3 and 5, those detector segments whose axis lies within the plane of the junction, likewise show no significant contrast at the junction. However, images 4 and 6 show very clear stripes of contrast variation in between the *p*-type and *n*-type regions. Moreover, this image contrast is completely reversed between the two images, a dark stripe in image 4 and a bright stripe in image 6. These results indicate that, at the interface between *p*-type and *n*-type regions, the incident electron beam is deflected away from segment 4 and towards segment 6. Since the direction of built-in electric field at a *p-n* junction should point from the *n*-type region to the *p*-type region, the observed direction of the electron beam deflection agrees completely with the expected direction of the built-in electric field. STEM imaging using a segmented type detector can thus visualize the built-in electric field of the *p-n* junction.

(The keen-eyed reader may detect very faint contrast present in images 1 and 3 of Fig. 2. This is attributed to residual defocus contrast — similar to Lorentz TEM — because the precise focus condition is harder to control under the objective lens off condition used to produce the narrow convergence angle of this experiment. Slight detector misalignment and inhomogeneity may also play a minor role.)

**Figure 3:**
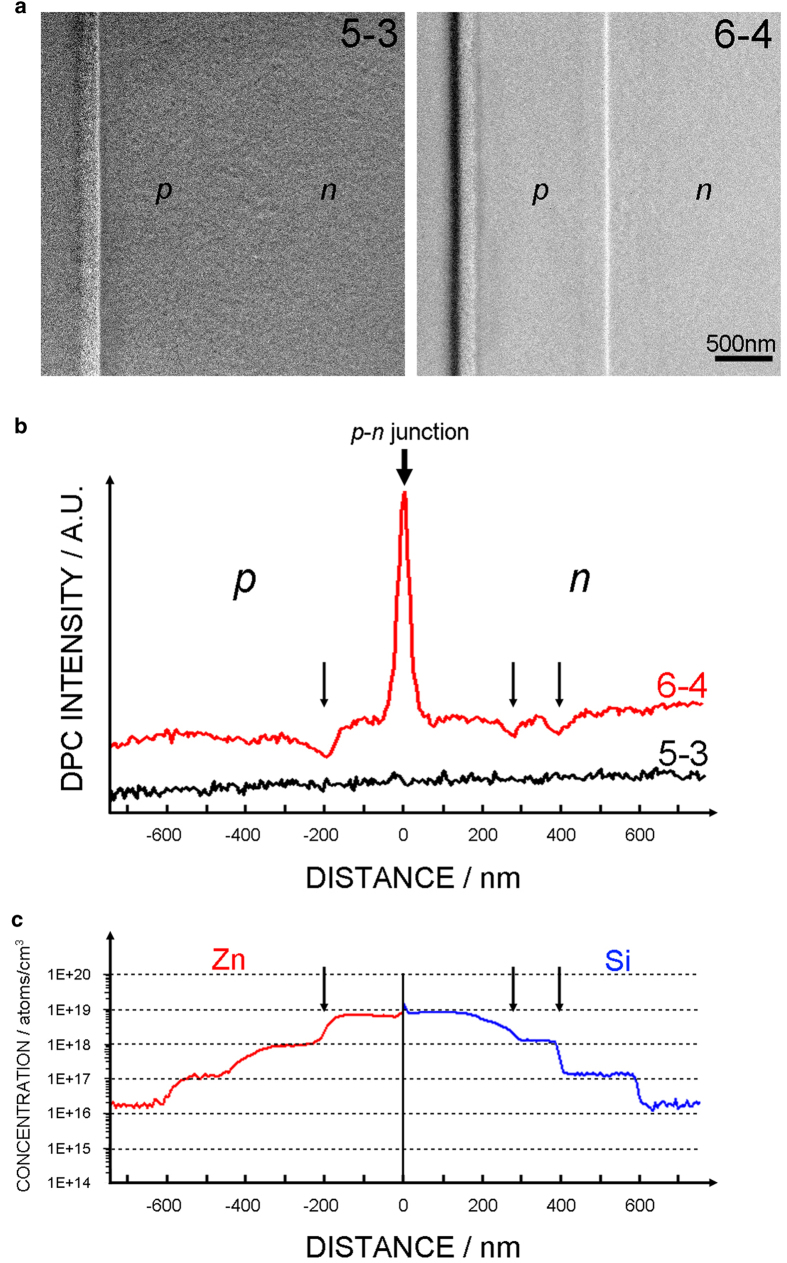
DPC STEM images of the *p-n* junction region in GaAs. (**a**) DPC STEM images of (5–3) and (6–4) using the segment images in Fig. 2. (**b**) The projected intensity profile of the DPC (5–3) and (6–4) images in the direction perpendicular to the *p-n* junction. (**c**) Dopant concentration profiles of Zn and Si in *p*-type and *n*-type regions, respectively, obtained by SIMS and adapted from ref. 16 with permission.

Figure 3a shows the DPC STEM images formed by subtracting the images from diametrically opposed segments shown in Fig. 2. Provided the probe size is smaller than the scale of the gradient in the electrostatic potential, the DPC image contrast derives from the beam deflection and is basically proportional to the magnitude of the electric field^10^,^14^,^15^. As we discuss later, this limit is not fully realized in the present experiment and some modest correction is therefore needed for quantitative analysis, but for qualitative interpretation the DPC STEM images can be taken as directly mapping out the electric field. The (6–4) image clearly visualizes the *p-n* junction, while the (5–3) image shows no significant image contrast at the *p-n* junction, showing again that the electric field direction is perpendicular to the *p-n* junction plane. To make this more quantitative, Fig. 3b shows the image intensity profile obtained by projecting the images in Fig. 3a along the vertical direction. The (6–4) signal shows a strong intensity peak. The (5–3) image shows no peaks of consequence.

In addition, weaker and opposite contrast stripes parallel to the *p-n* junction plane are visible within the *p*-type and *n*-type bulk regions in the (6–4) image in Fig. 3a, and more evident as dips in the (6–4) image intensity profile in Fig. 3b as indicated by the arrows. As shown in the schematic in Fig. 1, this sample has dopant concentration modulation at regular intervals in both *p-*type and *n-*type bulk regions in order-of-magnitude steps. These intervals were fabricated to be about 200 nm wide, but some deviation exists. Fig. 3c shows the dopant concentration profiles of Zn and Si in *p*-type and *n*-type regions, respectively, obtained by secondary ion mass spectrometry (SIMS)^16^. In the *p*-type region, the DPC image intensity dip corresponds well to the boundary between the 10^18^ and 10^19^ atoms/cm^3^ doped regions (~200 nm off from the *p-n* junction). In *n*-type region, the DPC image intensity dips can be found at ~280 nm and ~400 nm from the *p-n* junction, corresponding well to the boundary between the 10^18^/10^19^ and 10^17^/10^18^ atom/cm^3^ doped regions, respectively. Since these intensity dips are not found in the profile of (5–3) image nor in the profile of LAADF image (as shown in Fig. S6 in the supplemental material), these dips should not be caused by diffraction effects at the interfaces (e.g. from strain). Rather, these DPC image intensity dips can also be attributed to local electric field variation induced by the electrostatic potential step present at dopant concentration steps. Thus, DPC STEM can even sensitively detect the dopant concentration difference of an order of magnitude in GaAs semiconductor devices at higher dopant concentration regions. However, in the lower dopant concentration regions, we cannot clearly detect the DPC image intensity variation at the dopant concentration steps in either *p*-type or *n*-type regions. This is expected, since smaller concentration steps will produce a smaller local electric field variation which leads to smaller electron beam deflections and thus requires still higher sensitivity to detect. In addition, it has been shown that the inactive layers become thicker in lower dopant concentration regions in the same GaAs semiconductor specimen^16^, which would also reduce the projected local electric field variation. This could be due to the large surface depletion layer formed during the FIB sample milling.

## Discussion

**Figure 4:**
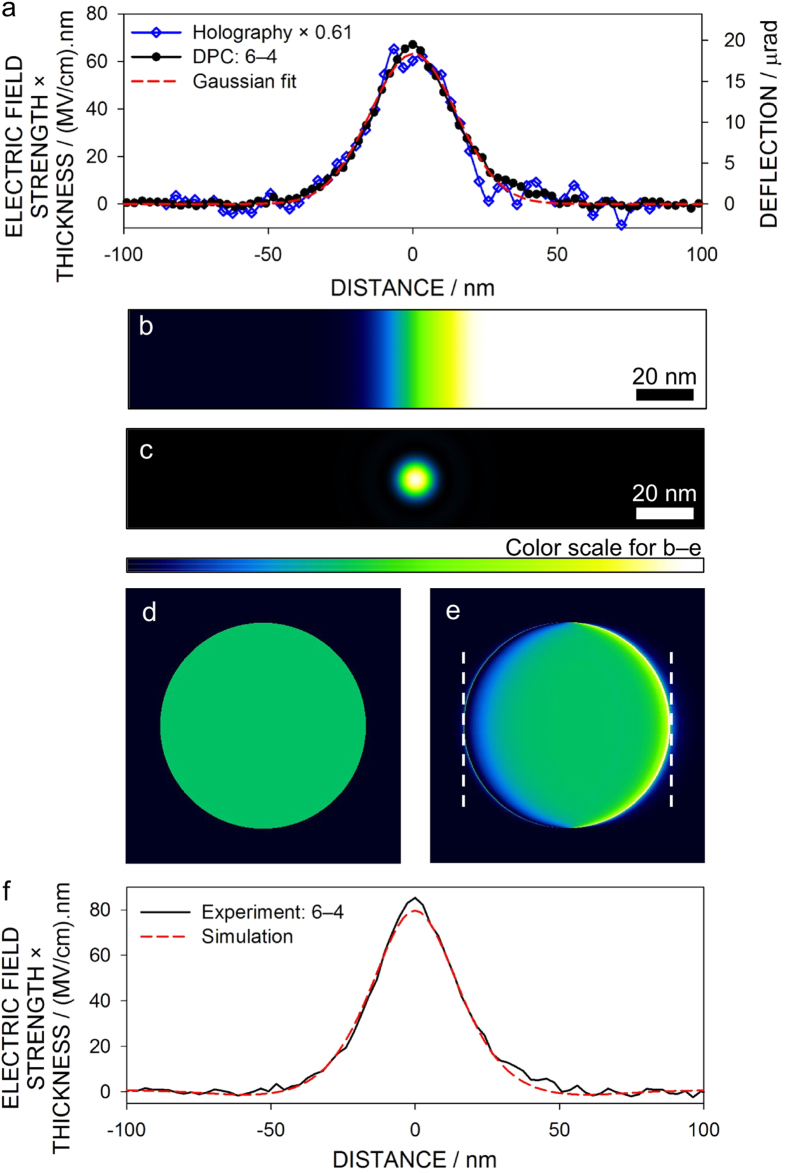
Quantitative analysis of the built-in electric field at the *p-n* junction from DPC STEM images. (**a**) Central portion of the DPC (6–4) image profile in Fig. 3(**b**), with vertical scales of electric field strength × thickness and deflection calibrated assuming rigid beam deflection (see supplemental material), together with similar data from electron holography^16^ and an empirical Gaussian fit. (**b**) Error function potential gradient and (**c**) STEM probe intensity distribution on the same length scale. (**d**) The diffraction pattern assuming constant potential. (**e**) The diffraction pattern assuming the potential and probe distributions from (**b**) and (**c**). (**f**) Comparison of the experimental DPC signal against simulation with calibration accounting for the intensity redistribution evident in (**e**).

To fruitfully explore new and unknown samples, the ability to qualitatively image the electric field structure during the experiment is vital to identify areas and features of particular interest, and DPC STEM live imaging enables this. However, for analysis purposes it is desirable to interpret the results quantitatively, to measure the (projected) electric field strength. To this end, the DPC signal was measured as a function of a calibrated deflection of the beam across the detector to establish a reference signal (see supplementary information for details). As a first pass analysis, assuming the potential gradient is constant over the area of beam illumination, this calibration can be used to convert the measured DPC profile in Fig. 3b into deflection units. The maximum electron beam deflection is determined to be 18.4 ± 4.7 μrad. This in turn can be converted into a product of the electric field strength and the thickness over which it acts. The result is shown in Fig. 4a, where the maximum electric field strength × thickness product is 63 ± 16 (MV/cm).nm. Fig. 4a also shows holography data for a very similar sample^16^. These data are noisier, because holography reconstructs the potential which must be differentiated to give the electric field profile. They have also been scaled, suggesting that the active thickness differed between the holography and DPC experiments which were not carried out on precisely the same region of the specimen. Nevertheless, the shape of the profiles is very similar.

To improve the quantitative analysis, an approximate form of the electrostatic potential distribution across the *p-n* junction is needed. The dashed line in Fig. 4a shows that, empirically, the DPC profile of the peak across the *p-n* junction is well approximated by a Gaussian profile. Simple electrostatic theory gives that the electric potential giving rise to a Gaussian variation in electric field strength is given by an error function distribution. Such a potential is shown in Fig. 4b, while the intensity distribution in the probe is shown on the same scale in Fig. 4c. Comparison of these figures calls into question the assumption that the potential gradient is constant on the scale of the probe. Fig. 4d shows the diffraction pattern for a constant potential while Fig. 4e shows the diffraction pattern assuming the probe and potential profiles in Figs. 4b,c and the phase object approximation. The resultant diffraction pattern shows a degree of deflection, but also a redistribution of the electron density not accounted for in the calibration analysis of Fig. 4a. As a second pass analysis, the calibration experiment is used to normalize experiment against simulation to account for the electron redistribution. Fig. 4f, which compares experiment and simulation on the same scale, shows a reasonable match assuming an error-function type potential of the form *V*_0_·erf(*x*/*d*) for a characteristic width *d* = 17 nm, and a peak electric field strength × thickness product of 80 ± 20 (MV/cm).nm, a modest correction over the first pass estimate.

We have given the electric field strength × thickness product, the quantity naturally determined by DPC STEM, electron holography or quantitative Lorentz TEM. Determining the electric field strength, the quantity of technological interest, is hampered because the measured total crystal thickness is not necessarily the active thickness: there may be inactive layers on the surfaces^16^. However, we can run the logic the other way, solving Poisson’s equations self-consistently based on the assumed dopant concentrations given in Fig. 3c to predict a peak electric field strength of 1.2 MV/cm , implying an active thickness of 67 nm. Since the total specimen thickness is 290 nm, this implies the inactive layer at each surface to be 112 nm thick, similar to previous findings^16^. Note that this 1.2 MV/cm electric field strength prediction assumes an ideal, very sharp *p-n* junction. In reality, however, there may be slight interdiffusion of the dopants across the junction, which would reduce the maximum field intensity. The estimated inactive layer thickness is therefore an upper limit. We also note in passing that because the measured quantity in the electric field strength × thickness product, variations can arise from changes in both the electric field strength and thickness. Thickness variation is evident in the LAADF image in Fig. S4b and in the slight incline in the “background” in the DPC signal in Fig. 3b. However, as can be concluded from Fig. 4a, thickness variation is insignificant over the width of the *p-n* junction.

In summary, we have demonstrated imaging of the built-in electric field in a *p*-*n* junction in a GaAs compound semiconductor by DPC STEM. The technique is further sensitive to the electric field variation at more modest dopant concentration steps. Model-based quantification of the field strength via the DPC signal at the *p-n* junction shows the technique can be used both qualitatively, for discovery, and quantitatively, for analysis. This ability to use STEM to directly probe local electric field variation at electrically important interfaces holds much promise for the simultaneous characterization of both the composition and the electromagnetic field distribution within materials and devices at nanometer dimensions.

## Methods

### D*PC STEM imaging*

DPC STEM images were taken with a 200 kV JEM-2100 F TEM/STEM electron microscope (JEOL Ltd., Tokyo, Japan) equipped with an aberration corrector (CEOS GmbH, Heidelberg, Germany). The instrumentation of the segmented detector used for this study has been reported elsewhere^17^, as have the details on the fabrication processes of the model GaAs *p*-*n* junction sample and the TEM sample preparation processes using focused ion beam (FIB) milling techniques^15^. The GaAs sample was characterized by electron holography and Lorenz TEM techniques prior to DPC STEM imaging. By comparing with these existing methods^18^,^19^, the capabilities and benefits of the present imaging method are independently verified. The sample thickness is estimated to be about 290 nm from convergent beam electron diffraction analysis. For an estimated electric field strength at the junction of 0.7 MV/cm, the nominal beam deflection would be 30 μrad. Because probe-forming convergence and detector collection angles cannot always be chosen to simultaneously maximize sensitivity, interpretability, and ease of quantitative analysis, some trade-off is usually required. Ideally, the probe should be significantly smaller than the scale of the electric field variation so as to undergo a simple deflection and not a more complicated redistribution, favouring large convergence angles. Deflection sensitivity, however, is set by the detector at a fixed fraction of the detector span and therefore favours smaller convergence angles. Whereas we previously achieved atomic-resolution DPC STEM imaging with a probe-forming semiangle of 23 mrad^11^, to obtain the deflection sensitivity needed to measure the small deflection expected here, the probe-forming aperture semiangle was set to 133 μrad and the microscope operated under objective lens off condition. The probe size, and thus the spatial resolution, is then about 12 nm. Note that the depth of focus of our STEM probe is very much larger than the sample thickness, which would ideally make the in-focus condition simple to achieve. However, under the operating conditions of the present experiment (objective lens off), setting the focus correctly is non-trivial, which sometimes results in a small, residual contrast in some images. The angle range from the optical axis of the quadrant detector segments used for DPC was correspondingly set to 108–162 μrad.

The specimen was initially oriented close to the [110] on-axis condition. However, as in the holography experiment, to reduce diffraction effects the sample is then tilted off-axis by a degree or two. The tilting condition was adjusted so as to maximize the contrast of *p*-*n* junction in live DPC STEM image, which we interpret to be the condition in which the incident electron beam is almost perpendicular to the electric field of the *p*-*n* junction. The almost identical width of the electric field profile between holographic and DPC imaging (Fig. 4a) implies highly comparable conditions, i.e. the tilt across the *p-n* junction is small.

## Additional Information

**How to cite this article**: Shibata, N. *et al*. Imaging of built-in electric field at a *p-n* junction by scanning transmission electron microscopy. *Sci. Rep.* **5**, 10040; doi: 10.1038/srep10040 (2015).

## Supplementary Material

Supplementary Information

Supplementary Movie
