# Supplementary material for: Imaging of built-in electric field at a p-n junction by scanning transmission electron microscopy
Source: Sci Rep. 2015 Jun 12;5:10040. doi: 10.1038/srep10040 (PMC4464396; doi:10.1038/srep10040)
Supplement: Supplementary Information [file srep10040-s1.pdf]

Imaging of built-in electric field at a *p-n* junction by scanning transmission electron microscopy

N. Shibata, S.D. Findlay, H. Sasaki, T. Matsumoto, H. Sawada, Y. Kohno, S. Otomo, R. Minato and Y. Ikuhara

Quantifying the projected electric field strength from the differential phase contrast signal

The differential phase contrast (DPC) signal maps the relative deflection, or more generally the relative redistribution, of the diffraction pattern intensity as the probe is raster scanned across the sample. Given the estimated sample thickness of 290 nm, the first step towards quantitative analysis of the DPC signal is to rule out, as best we may, the possibility of confounding influences from multiple scattering of the electrons. In particular, we must establish that the bright field disk, that is to say the unscattered or "forward scattered" portion of the diffraction pattern, is well-defined and has uniform intensity as this was assumed in the analysis.

The specimen was tilted a degree or so away from the exact [110] zone-axis orientation for two reasons. The first reason is to maximize the DPC signal: if the simple field-induced deflection interpretation holds, this accommodates the possibility that the exact [110] orientation does not quite lie in the plane of the *p-n* junction. Though the focused ion beam prepared specimen is expected to yield high thickness uniformity, in some orientations bend contour contrast was evident in the bright field and DPC images. The second reason is therefore to find an orientation in which the "background signal" is constant, i.e. bend contours are minimized. This is interpreted to correspond to reduced dynamical scattering effects, circumstances one might be cautiously optimistic of yielding a uniform bright field disk.

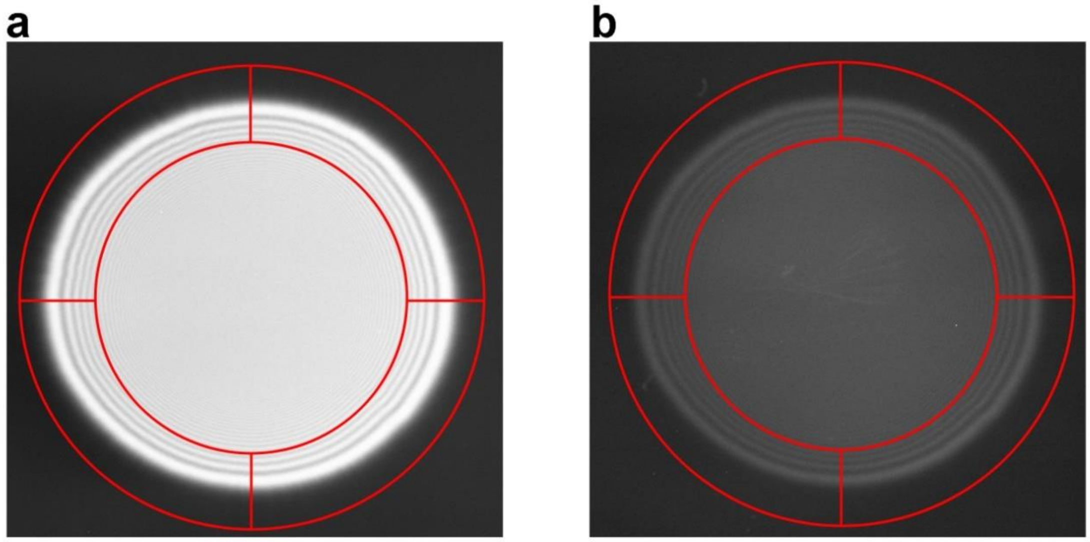

Fig. S1. Bright field disk (a) without and (b) with the sample present, recorded via photographic film and scanned on a linear scale. Exposure times of the photos are (a) 1 s and (b) 16 s. Though scattering by the sample has reduced the intensity in the bright field disk, the disk itself is still clearly defined. Moreover, the intensity distribution inside the segmented detector regions used for DPC imaging (indicated by the red lines) is quite uniform with the sample present.

Fig. S1 shows images of the bright field disk (a) without and (b) with the sample present recorded via photographic film[1]. The signal in the latter is significantly weaker than in the former, evidence that a significant amount of scattering takes place. Some faint contrast variation can be seen around the center of the bright field disk. However, in the regions used for the DPC imaging, highlighted by the red lines, the intensity distribution in the bright field disk is quite uniform (the fine fringing present being basically rotationally symmetric). Thus, our strategies to orient the sample can indeed

---

[1] No CCD camera was available in the microscope on which our experiments were carried out. Ultimately, the segmented detector approach to DPC imaging presented here may be superseded by pixel detectors (e.g. direct detection cameras). Proof-of-principle datasets along these lines already exist [S1], but, so far as we are aware, no such detector has yet achieved DPC “live imaging”.

produce uniform bright field disk intensity. This is consistent with Fig. 2 of the manuscript, which shows that there is no significant isotropic signal at the *p-n* junction location (cf. at the specimen surface). Therefore, the highly directional redistribution evident in detectors 4 and 6 but not 3 and 5 is attributed to a long range (relative to the atomic scale) electric field present at the *p-n* junction, rather than scattering from either the crystalline structure or from local disorder.

It remains important to establish that such conditions are common enough for this imaging strategy to be broadly applicable and whether they can be reliably found using a segmented detector. To explore this, Fig. S2 plots channeling maps for plane wave incidence and a point, on-axis detector as a function of sample orientation about the [110] orientation. For any disk-like region with angular diameter smaller than the smallest crystal reciprocal lattice vector, such as the 133 μmrad radius probe-forming aperture angle of the present experiment, the intensity distribution in these maps can be interpreted as that in the bright field disk due to elastic scattering at that orientation. The fine structure clearly visible in the channeling maps extends down to this length scale (the red circles in the enlarged images in the bottom right of Fig. S2 indicate the size of the bright field disk used in the present experiment). Sample orientations for which the bright field disk would not be uniform are clearly possible.

To better quantify this, Fig. S3(a) plots a measure of the intensity variation (specifically the standard deviation divided by the mean, σ/μ) within a bright field disk of radius 133 μrad as a function of specimen orientation, for a sample thickness of 290 nm. The black and deeper blue regions are those where the intensity distribution within the bright field disk is quite uniform. The brighter colored regions are those for which the intensity variation within the bright field disk is considerable.

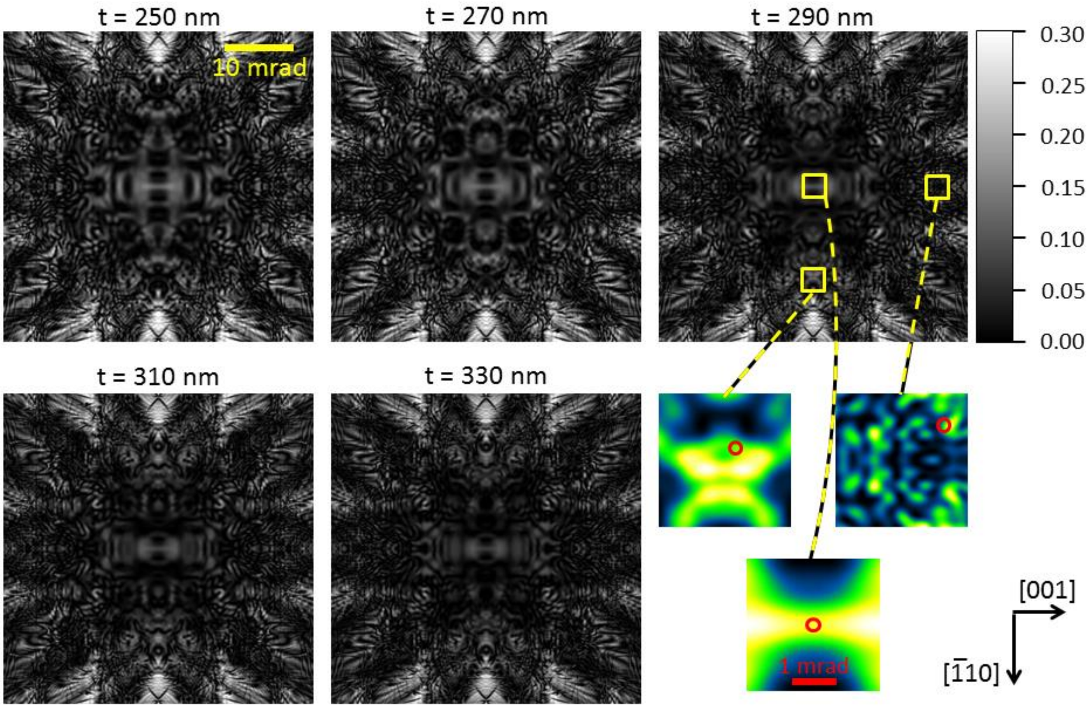

Fig. S2. Channeling maps: the intensity obtained assuming plane wave incidence and an on-axis point detector as a function of specimen orientation with the exact [110] zone axis in the centre. Circular patches of these images with angular diameter smaller than the smallest crystal reciprocal lattice vector can be interpreted as the intensity in the bright field disk for a convergent probe of that same convergence angle. The colored images are magnified extracts from the 290 nm thickness map, and are shown on their own intensity scales. The red circles in this image denote the size of the bright field disk in the present experiment.

Direct imaging of the bright field disk is the best way to assess whether or not the bright field disk has a uniform intensity distribution. Another piece of evidence for the presence of dynamical scattering effects is that, despite the focused ion beam sample preparation being expected to yield a sample of fairly uniform thickness, in some orientations bend contours were clearly visible in the bright field and DPC live images. In seeking an orientation giving a reasonably uniform intensity bright field disk, we were guided by the assumption that orientations for which the bright field or DPC live images did not show such bend contours were those where the effects of channelling were minimized. Fig. S3(b) plots the thickness derivative of the channeling map (normalized by the intensity at each orientation) from Fig. S2 for the case t = 290 nm. It

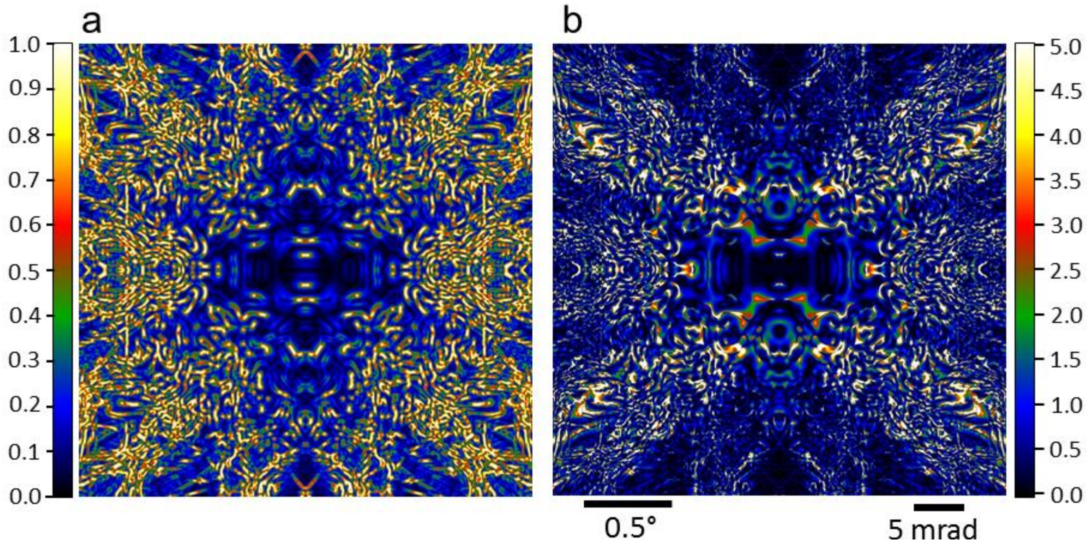

Fig. S3. (a) Variation in bright field disk intensity distribution measured by intensity standard deviation divided by intensity mean assuming a bright field disk of radius 133 μrad. (b) Normalized thickness variation in intensity ($(\Delta I/\Delta z)/I$) of the forwards scattered electrons. Both maps assume 290 nm of GaAs and are shown as a function of specimen orientation centered about the [110] direction.

is seen that there is a strong – though not complete – correlation between those orientations for which the bright field disk intensity is quite uniform, the dark portions of Fig. S3(a), and those for which the bright field intensity is least sensitive to variations in specimen thickness, the dark portions fo Fig. S3(b). This supports seeking specimen orientations that eliminate bend contour contrast in the bright field image as a useful heuristic for predicting orientations in which the intensity distribution within the bright field disk might be uniform and shows another advantage of the DPC STEM live imaging.

To quantify the strength and distribution of the projected built-in electric field we must quantitatively interpret the magnitude of the DPC signal in terms of the redistribution of electron density in the detector plane. By displacing the bright field disk in known steps across the surface of the detector, we obtain a calibration curve of DPC signal

against displacement.[2] Results are shown in Fig. S4 for three data sets recorded from the same sample but in different microscope sessions on different days. The reproducibility of the experiment is well within the error bars on the data (which derive from intensity fluctuations in the images attributed primarily to Poisson counting statistics and could therefore be reduced by increased recording time).

It is seen that the change in DPC intensity is essentially linear with shift for shifts of up to 20 μrad. This is expected. The radius of the bright field disk is 133 μrad while the inner radius of detector segments 4 and 6 is 108 μrad. For displacements smaller than

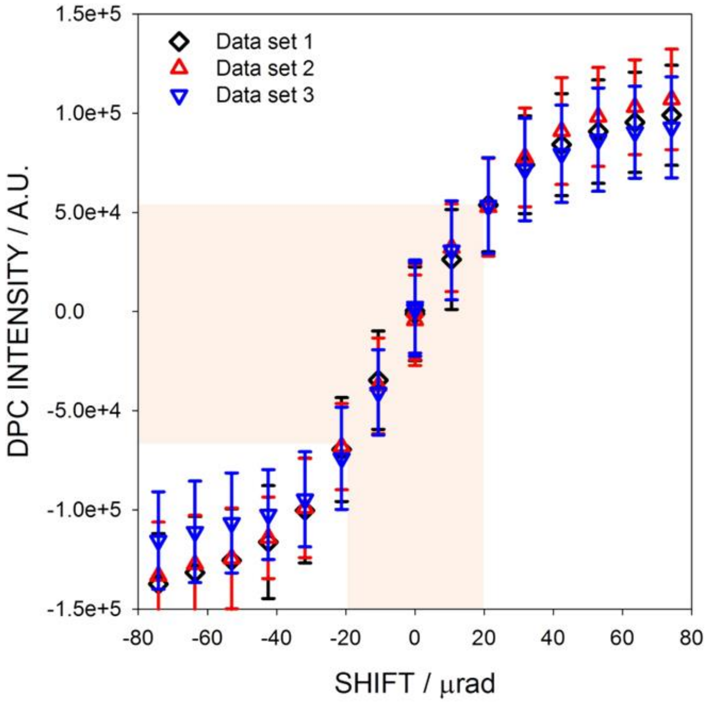

Fig. S4. DPC intensity (6–4) as a function of shift of the diffraction pattern across the detector. The different data sets were obtained from the same specimen but during different experiments. The shading is a guide-to-the-eye of the range over which the DPC intensity is approximately linear with shift.

---

[2] Given the intensity difference with the sample present as seen in Fig. S1, the calibration is done with the sample in place. The more indirect approach in which the sample is absent serves to increase the error bars through the additional processing steps but does not significantly change the results.

20 μrad, displacement of the bright field disk towards segment 4, say, increases the intensity of segment 4 and decreases it on segment 6 in approximately equal measure, and the DPC intensity includes both changes.[3] However, for large enough shifts – specifically, shifts beyond 25 μrad – the bright field disk ceases to fall on segment 6 at all, and all change in the DPC intensity becomes due to the intensity change on segment 4 alone, changing the functional dependence of the DPC intensity with shift. This limitation could be to some extent overcome by further using the segments both within and beyond those in the 108–162 μrad ring or via changes in camera length. The inherent limitations in the fixed geometry of this segmented-type detector should be fully overcome as pixelated detectors become available [S1]. However, as we will proceed to show, the DPC signal of the *p-n* junction interpreted as a pure shift would lie within the linear region. (As per Fig. 4(e), the effect of the field is not so much a deflection as an intensity redistribution, however since this effect is overwhelmingly confined within close proximity to the edges of the bright field disk, the analysis still holds.)

We will focus on data set 1, since it was recorded in the same session as the DPC images shown in the main manuscript. Fig. S5 reproduces the approximately linear region of that data and shows the line of best fit

$$I = m\,s + c \qquad -\,(1)$$

where for I denoting the DPC intensity in the microscope "units" given and s denoting the shift in μrad we find

$$m = (2.9 \pm 0.7) \times 10^3 . \qquad -\,(2)$$

---

[3] The curved boundaries of the bright field disk and the detector segments mean that linearity is not exact, but it is a good approximation.

From the Gaussian fit to the projected DPC (6–4) image profile in Fig. 4(a), the change in DPC intensity from the bulk to the peak at the *p*-*n* junction is ΔI = 5.3×10^4. It follows from equations (1) and (2) that this corresponds to a relative shift of Δs = 18.4±4.7 μrad. Note that the implied shift is within the 20 μrad range of validity of the approximately linear treatment.

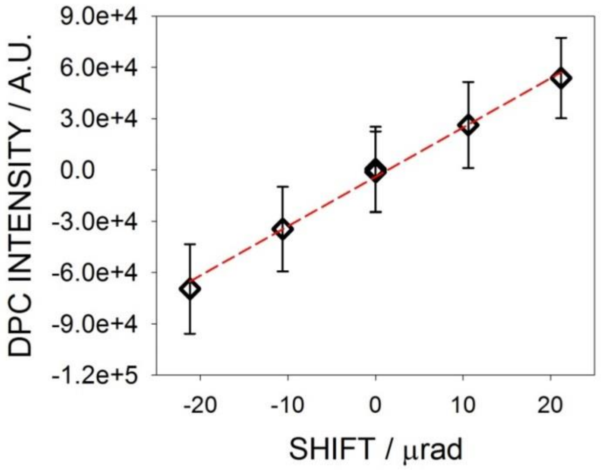

Fig. S5. DPC intensity (6–4) as a function of shift within the approximately linear response region for data set 1. The dashed line shows the linear fit.

Assuming a uniform electric field within the sample, the relationship between the angular displacement, the electric field and the sample thickness is [S2]

$$\gamma = \frac{t}{2K^2}\frac{2m^*e}{h^2}E' \approx 0.29E't. \qquad -(3)$$

where *t* is the specimen thickness, *K* is the wavevector, *m** is the relativistic mass, *e* is the magnitude of the charge of the electron, *h* is Planck's constant, *E'* is the electric field strength, and in the final step we assume 200 keV electrons, γ in μrad, *t* in nm and *E'* in MV/cm. Using the estimated deflection angle of 18.4 μrad we obtain a peak electric field strength × thickness product of 63±16 (MV/cm).nm. This is the basis of the "first

pass estimate" in the main manuscript.

The above analysis assumes that the electric field leads to a simple shift, a rigid displacement of the diffraction pattern across the detector, which is true for an electric field which is uniform on a much larger scale than the width of the probe. However, in the present data the full-width-half-maximum of the Gaussian fit to the DPC profile (Fig. 4(a)) is 35 nm while the width of the probe intensity is about 12 nm, of comparable scale. Landauer et al. [S3] have proposed a more general reconstruction algorithm from DPC signals to scattering potential, but only in the framework of the weak phase object approximation. Using our first pass estimate and assuming an active thickness of 67 nm, a width of 10 nm implies a potential difference of around 1.2 eV, which for a 67 nm active region thickness implies a phase accumulation of about 0.6 rad, which is not necessarily "weak". A more general reconstruction is possible in the case of a so-called first moment detector [S4]. The extent to which a segmented detector approximates that ideal case is an area of ongoing investigation, but currently remains unclear. Instead, we proceed as follows.

In the phase object approximation, justified by the weak potential profile and very narrow probe-forming convergence angle, we may readily simulate the electron redistribution for any given test model potential. For a suitably parameterized profile, we use trial and error[4] to obtain a good match with the observed DPC intensity profile. Since empirically we found a Gaussian profile to be a good fit to the DPC intensity profile, let us take an error function as a simple, two-parameter ansatz for the potential profile, i.e.

---

[4] More rigorously one would use a fitting procedure, but within the errors present here trial and error suffices.

$$V(x) = V_0 \operatorname{erf}\left({}^{x}/_{d}\right), \qquad -(4)$$

since it may readily be shown that the corresponding electric field profile is Gaussian,

$$E(x) = \frac{2}{\sqrt{\pi}}\frac{V_0}{d}e^{-x^2/d^2}. \qquad -(5)$$

Simulation is normalized against experiment by the expedient of simulating the experiment in Figs. S4 and S5 and determining the normalization constant between them, which procedure inherits the 25% relative error of equation (2). Trial and error lead to the match shown in Fig. 4(f), obtained for d = 17 nm and a peak electric field strength × thickness product of 80±20 (MV/cm).nm.

It will be noted that the experimental DPC intensity profile has a slightly higher, sharper peak and perhaps slightly broader tails than does our trial-and-error simulation. Given the present error in normalizing the experiment against the simulation (about 25%), we see no meaningful advantage in attempting a more elaborate ansatz for the potential. Ultimately, it is desirable to develop a robust method for reconstructing the sample potential from the signals on a segmented detector given strong (at least relative to the weak phase object approximation) scattering conditions.

Image intensity profile across the *p-n* junction of LAADF STEM image (image 2)

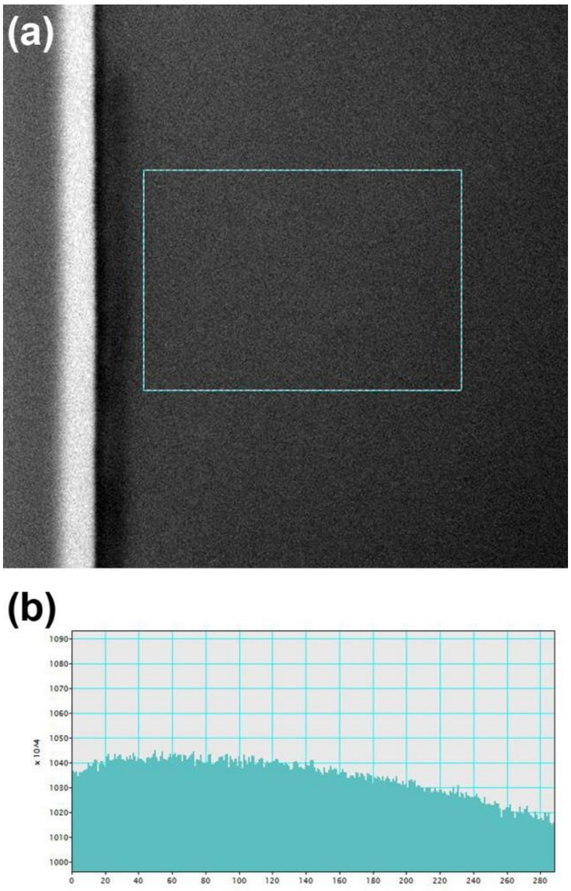

Fig. S6. (a) LAADF STEM image (image 2 in Fig. 2) and (b) the corresponding image intensity profile across the *p-n* junction. The intensity profile is summed along the vertical direction within the rectangular region shown in (a).

Description of the supplemental movie S1

The supplemental movie shows the live observation of the *p*-*n* junction using our DPC STEM system. The upper two panels are two DPC STEM images showing the in-plane electric field components in the two perpendicular directions. The lower left panel shows the electric field color map (a color wheel indicating the electric filed strength/direction is shown below) calculated from the two DPC STEM images. The lower right panel shows the electric field strength map calculated from the two DPC STEM images. The vertical strong line contrast seen at the center of the images corresponds to the *p*-*n* junction.

The observing condition for the live DPC STEM here (relative orientation between the detector and the sample) is not the same as the static observation shown in the main manuscript. In this case, the left hand side of the *p*-*n* junction is the *n*-type region and the right hand side is the *p*-type region.

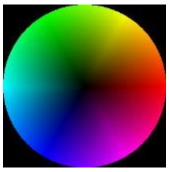

References

[S1] T.J. Pennycook, A.R. Lupini, H. Yang, M.F. Murfitt, L. Jones, P.D. Nellist, Ultramicroscopy, in press, doi:10.1016/j.ultramic.2014.09.013.

[S2] L. Reimer, *Transmission Electron Microscopy 4th Edition* (Springer-Verlag) (1997)[S3] M.N. Landauer, B.C. McCallum, J.M. Rodenburg, Optik, **100**, 37 (1995).

[S4] E.M. Waddell, J.N. Chapman, Optik, **54**, 83 (1979).
